# Predicting hazardous drinking in late adolescence/young adulthood from early and excessive adolescent drinking - a longitudinal cross-national study of Norwegian and Australian adolescents

**DOI:** 10.1186/s12889-019-7099-0

**Published:** 2019-06-21

**Authors:** Frøydis Enstad, Tracy Evans-Whipp, Anne Kjeldsen, John W. Toumbourou, Tilmann von Soest

**Affiliations:** 1 Division of Mental and Physical Health, Department of Child Health and Development, Norwegian Institute of Public Health, PO Box 222 Skøyen, N-0213 Oslo, Norway; 2 Centre for Adolescent Health, Murdoch Children’s Research Institute, Parkville, Victoria Australia; 3 Department of Paediatrics, The University of Melbourne, Parkville, Victoria Australia; 4 Bjørknes University College, Oslo, Norway; 5 Deakin University, Geelong, Victoria Australia; 6 PROMENTA Research Center, Department of Psychology, University of Oslo, Oslo, Norway

**Keywords:** Early onset of drinking, Early onset of excessive drinking, Prospective study, Hazardous drinking, Adolescence, Late adolescence, Young adulthood, Cross-national study

## Abstract

**Background:**

Research has consistently shown that early onset of drinking (EOD) is associated with alcohol-related problems in adulthood. However, recent reviews have identified several limitations in the early onset literature, including the use of retrospective reports, insufficient control for potential confounders, ambiguous definitions of the concept, and an assumption that early onset is independent of cultural norms and national alcohol policies. This study addresses these limitations by examining whether EOD, independent of early onset of excessive drinking (EOE), prospectively predicts hazardous drinking in late adolescence/young adulthood in Norway and Australia, two countries with different drinking cultures.

**Methods:**

Data were drawn from two population-based longitudinal studies; the Norwegian Tracking Opportunities and Problems Study (*n* = 329) and the Australian International Youth Development Study (*n* = 786). Data were collected prospectively from mid adolescence (14–16 years) to late adolescence/young adulthood (18–25 years) and a modified Poisson regression approach was used to estimate prevalence ratios. Adolescent self-reports included measures of EOD and EOE. Young adults completed the Alcohol Use Disorders Identification Test (AUDIT). The results were adjusted for adolescent factors; age, gender, impulsivity, hyperactivity, conduct problems, smoking, early sexual intercourse and friends’ substance use, and family factors; alcohol and drug use in the family, maternal education, family management and monitoring.

**Results:**

Hazardous drinking was identified in 46.8 and 38.9% of young adults in Norway and Australia, respectively. Both EOD and EOE in adolescence were significantly related to an increased risk of alcohol-related problems in late adolescence/young adulthood in both studies, even when adjusting for possible confounders.

**Conclusion:**

Our findings indicate that adolescent drinking behaviour is an indicator of alcohol-related problems in late adolescence/young adulthood, even when controlling for a variety of covariates. This finding is in contrast to previous research on older adults, where no association between adolescent drinking and later alcohol-related problems were found when controlling for covariates. The divergence in findings may suggest that the impact of EOD/EOE is limited to the late adolescent and young adult period. Preventing drinking in early adolescence may thus have some impact on the drinking patterns in late adolescence/young adulthood.

## Background

Empirical studies have consistently shown that early onset of drinking (EOD) is related to later adverse alcohol-related outcomes [1–16]. Many of these studies suggest a causal relationship between EOD and subsequent problems [1–6, 10, 14]. However, recent reviews of the literature have questioned the assumption of a causal link [17–19]. In particular, it has been noted that: (a) research on EOD and its association with future adverse outcomes is largely based on adult samples asking participants to recall retrospectively their age of drinking onset, potentially introducing recall biases; (b) the few longitudinal studies measuring drinking onset prospectively typically suffer from lack of control for important covariates that may serve as confounders, such as externalising behaviours; (c) most studies do not provide information about whether having consumed small amounts of alcohol at an early age are related to adverse outcomes, or whether such associations are first seen when more substantial amounts are consumed; and (d) the association between early onset and adverse outcomes may vary according to the cultural context [17, 18, 20]. By using longitudinal data from two different countries, this study aims to provide a better understanding of the relationship between early drinking behaviours and hazardous drinking in late adolescence/young adulthood.

As thoroughly demonstrated in a review by Kuntsche et al. [17] the literature in this field suffers from poor reliability of the early drinking measurements as many studies are based on retrospective reports introducing potential bias. The concerns are related partly to the tendency of forward telescoping [21], as respondents tend to report the age of onset closer to their current age when interviewed. Moreover, heavy drinkers are to a greater degree biased to report an early onset of drinking than light drinkers when age of drinking is assessed retrospectively [22]. The association between retrospectively reported onset of drinking and adult drinking behaviour may thus be explained by methodological artefacts. It has therefore been argued that prospective studies from adolescence, close in time to when onset typically occurs, are more suited to examine EOD and its relation to later alcohol-related problems [16]. Furthermore, individual level variables (e.g., conduct problems, impulsivity-hyperactivity, antisocial peer environment, health risk behaviour), and family level variables (e.g., alcohol and drug use in the family) need to be included as covariates. Developmental theories have pointed to their relevance for the development of substance use disorders [23] and empirical research link these same factors to early onset of alcohol use [3, 8, 16, 24]. Such factors are potential confounders, as they may reflect shared vulnerability to both early onset and adult alcohol-related problems.

Our review of the literature identified 15 prospective studies addressing the impact of EOD on later drinking patterns or alcohol-related problems. Among these, four followed the participants only to mid adolescence [7, 25–27] and five studies did not control for important confounding variables, such as conduct problems or parental alcohol and substance use [3, 14, 28–30]. Results from the remaining six studies following participants from early adolescence to adulthood, and with comprehensive control for possible confounders, were mixed: Whereas four studies found that the association between EOD and adult alcohol-related problems was completely explained by relevant covariates [16, 31–33], two other studies found that the association between EOD and heavy alcohol consumption remained, even after comprehensive control for potential explanatory factors, including conduct problems and parental drinking patterns [4, 11]. Thus, despite the growing number of prospective studies in this field, findings are still diverging, which may be related to varying operationalisations of early drinking by researchers, and different cultural contexts across studies.

Most of the studies in the early onset literature conceptualize onset as having had more than a few sips on one or more occasions [1, 4, 6, 14, 15, 27]. However, scholars have recently challenged the notion that consuming small amounts of alcohol in itself should have such profound impact on adolescent development [17, 34]. If the association between early drinking and adult alcohol-related problems is based on biological mechanisms, for example by early alcohol use affecting the cognitive functioning of the brain (i.e. [35]) or disrupting maturational processes [36], it is more plausible to assume that such changes would occur only if the amount of alcohol consumed had a potential neurotoxic effect. Likewise, if the mechanism is of a social nature, for example through changing the identity or social role of those drinking early (i.e. [14]), one could assume that such changes would be triggered only if the amount consumed violates a cultural expectation of acceptable behaviour. If the adolescents’ first encounter with alcohol is in a cultural setting where moderate alcohol use is the norm, e.g., a family dinner, it may be rather improbable that such behaviour would trigger a different developmental trajectory. Underage excessive drinking on the other hand, is a more clear breach of socially accepted behaviour, and as such has more potential to trigger a change in identity or role. A recent review supports this explanation, concluding that there are larger and more important effects observed in relation to the onset of regular drinking and experiences of intoxication than age of first drink [18].

To our knowledge, only two longitudinal studies have directly addressed whether EOD independently of early onset of excessive drinking (EOE) serves as a risk factor for later alcohol problems. First, Warner and White [33] found that the only onset related variable associated with adult alcohol problems, after a rigorous control of possible confounders, was feeling drunk at initiation. This study however only addresses different aspects of the very first drinking episode (i.e., in or outside a family context, early versus late onset, experience pleasure or not, and feelings of drunkenness). Second, Morean et al. [12] examined age of onset and delay to first intoxication as independent predictors of heavy drinking in college students. The results showed that both an early onset and a short delay independently predicted heavy drinking, indicating that EOE confers unique risk relative to EOD, but that both factors are related to increased risk. However, the study was based on retrospective reports of drinking onset and did not control for indicators of externalizing problems or other risk taking behaviour. Thus, knowledge on whether EOE foreshadows a different drinking pattern in young adulthood from EOD, or whether any early exposure to alcohol is detrimental, is limited.

Cultural norms and national alcohol policies may also be of importance in understanding the relationship between early drinking behaviours and adult alcohol use [37]. Whilst excessive drinking and drunkenness in adolescents is likely to be viewed as a social problem in most cultures, the degree to which low levels of adolescent drinking, particularly within adult-supervised contexts, is viewed as problematic varies largely across countries and cultures. Research has shown that in permissive cultures where moderate drinking among adolescents is culturally normative, EOD is to a lesser degree linked to other markers of deviance, such as conduct problems, than in cultures were EOD is considered a greater social problem, such as the United States, where most of the research on EOD has been conducted [20]. Underage excessive drinking, on the other hand, may be more consistently related to negative outcomes across cultures. Although EOD and EOE take on meaning in the larger cultural and political context, no previous study has applied a cross-national design when examining possible associations. The present study uses data from Norway and Australia, countries with different alcohol policies and drinking cultures.

Of particular interest, prevention strategies aimed at youths differ considerably in the two countries. Norway has one of Europe’s most restrictive alcohol policies [38] with an emphasis on reducing use through reducing the demand and availability [39]. As a consequence, underage drinking is illegal with no exceptions, and the prohibition also applies to suppling minors with alcohol. Information material aimed at youth highlights activities to increase factual knowledge about alcohol, strengthening the individual’s ability to resist peer pressure and facilitating get-togethers where no alcohol is served [40]. In contrast, the overarching goal of Australia’s strategy during the study period was to minimize harm associated with alcohol use [41]. Information strategies aimed at adolescents included advice on how to stay safe and ‘in control’ when drinking [42]. The per capita consumption in Norway is among the lowest in the Western countries both among adolescents and in the adult population [43, 44], whereas Australia is among the highest among the adult population [44]. The prevalence of heavy episodic drinking is about the same in the two countries. However, somewhat paradoxically, the Norwegian drinking pattern, i.e., how they drink rather than how much, is characterized as more risky than the Australian [44]. Cultural attitudes towards underage drinking may influence associations between early drinking behaviours and later detrimental outcomes. One could hypothesize that the relationship between EOD and later problem drinking is weaker in Australia than in Norway, because EOD in Norway may represent a greater violation of formal rules and as such is more closely related to other problem behaviours which are associated with hazardous drinking in adulthood.

In summary, the present study’s primary aim is to examine the association between EOD and the association with risk of alcohol-related problems in late adolescence/young adulthood. In particular, this study addresses several limitations of most previous research by examining (a) whether EOD (14–16 years) prospectively predicts risk of alcohol-related problems in late adolescence/young adulthood (18–25 years), (b) whether this relationship remains upon adjustment for a comprehensive number of covariates that may function as confounders, (c) whether early onset of more than a few sips of alcohol is predictive of later alcohol problems or whether only the consumption of more excessive amounts of alcohol in early age is related to such risk, and (d) whether relationships between EOD and subsequent risk of alcohol-related problems are similar in samples drawn from two different countries with different alcohol policies and drinking cultures.

## Methods

### Study design and setting

Data were obtained from the Tracking Opportunities and Problems Study (TOPP) and the International Youth Development Study (IYDS); two independent longitudinal samples of adolescents and young adults in the eastern part of Norway (henceforth termed NOR) and Victoria, a southern state in Australia (henceforth termed AUS), respectively.

The participants in TOPP were recruited in 1993 when the families attended their toddlers’ 18 month vaccination at child health clinics. Originally 1081 families from 19 geographical health care districts in eastern Norway (28% living in large cities, 55% in densely populated areas and 17% in rural areas), were invited to the study, of whom 86.9% participated in the first wave. Details of the study are described elsewhere [45]. Data used in this paper were obtained from surveys of the participants and their mothers conducted in 2006 (t6; henceforth termed adolescence, median age = 14.6, range 14.0–15.8) and 2011 (t8; henceforth termed late adolescence/young adulthood, median age = 18.9, range 18.3–19.9). The overall response rate at t6 and t8 was 49% (*n* = 458) and 47% (*n* = 441) respectively, based on participants at t1 (*n* = 939, 87% of the eligible sample). The final sample, with data at both time points of interest were available for 329 young people (131 males and 198 females). Thus, from the 1081 families invited to the study, 30.4% were included in the present study. Previous attrition analyses have shown that dropout was predicted by low maternal education level and being male [46, 47]. The study was approved by the Regional Committees for Medical and Health Research Ethics.

The IYDS is an ongoing binational longitudinal study initiated in 2002 of three cohorts (t1 in grade 5, grade 7 and grade 9) of young people in Victoria, Australia and Washington State, United States. This study uses data from the Australian sample only as data on young adults in the US was not available at the time of analysis. The oldest age (grade 9) cohort was used as this best matched the ages at which TOPP data collection was conducted. The IYDS is a school-based survey with a state wide representative sample recruited and surveyed in 2002 and followed up to 8 subsequent waves. Details of the study and school recruitment procedures has been described elsewhere [48]. In the IYDS oldest cohort, 1288 students were eligible to participate, of whom 973 (76%) participated at t1 and 788 in the follow-up (81% retention). Honesty criteria based on responses to the t1 survey was used to remove 2 participants and so the final sample with data at both time points was 786. The surveys included in this study were conducted in 2002 (t1; henceforth termed adolescence, median age = 14.9, range 13.8–16.2) and 2010 (t7; henceforth termed late adolescence/young adulthood, median age = 22.9, range 21.6–24.6). The IYDS also includes mother report on education level from a phone survey conducted in 2002.

### Measures

In NOR and AUS, *hazardous drinking* in late adolescence/young adulthood was assessed using the Alcohol Use Disorders Identification test (AUDIT) [49]. The AUDIT is a widely used 10 item screening tool to identify persons with hazardous (i.e., those who are at risk of alcohol-related problems) and harmful (i.e., those experiencing some alcohol-related problems) patterns of alcohol consumption. The scores of the AUDIT range from 0 to 40, and the generally accepted cut-off for hazardous drinking is 8 and above, and a cut-off for harmful use is 16 and above [49–51]. The present study examines only hazardous use. In the NOR questionnaire, Item 3 differs from the original AUDIT in that it asks “How often do you have five or more drinks on one occasion” instead of six. Cronbach’s alpha for the whole scale was .77 (NOR) and .83 (AUS). In the NOR sample, drinking smaller amounts of alcohol and excessive use in adolescence were measured by adolescent self-report using two items; “Have you ever tasted more than a few sips of alcohol?” and “During the past 12 months, have you had so much to drink that you felt clearly intoxicated?” with five response categories (Never, Once, 2–5 times, 6–10 times and, More than 10 times). Responses were reclassified into two dummy variables; EOD relative to abstinent (have tasted one or more times, but not been intoxicated past 12 months, EOE (tasted one or more times and been intoxicated one or more times past 12 months). In the AUS sample these concepts were measured by adolescent self-report using three items; “In your lifetime, have you ever had more than just a few sips of an alcoholic beverage (like beer, wine or spirits)?” with five response categories (Never, 1 or 2 times, 3 to 5 times, 6 to 9 times, 10 or more times), “Think back over the past 2 weeks. How many times have you had five or more alcoholic drinks in a row?” (None, Once, Twice, 3–5 times, 6–9 times, 10 or more times) and “How often over the past year has your alcohol use caused you to get so drunk you were sick or passed out?” with items rated on an 8-point scale from “never” to “40 times or more”. Responses were reclassified into two dummy variables; EOD relative to abstinent (one or more times of lifetime alcohol use, but never binged and never sick or passed out), EOE (one or more times of lifetime alcohol use and one or more times binged and/or one or more times sick or passed out).

*Hyperactivity* (NOR) was measured with a subscale of the Strengths and Difficulties questionnaire (SDQ) [52] with five items (sample items: “I am restless”, “I find it hard to sit down for long”, “I think before I do things”) with four response categories ranging from 3 (Fits very well) to 0 (Doesn’t fit at all) (Cronbach’s alpha = 0.71). *Impulsivity* (AUS) was measured by three items describing typical ways to act (i.e., thinking before acting, rushing into things, answering before thinking). Response options were NO! (1), no (2), yes (3), YES! (4) (Cronbach’s alpha = 0.57, Cronbach’s alpha based on polychoric correlation = 0.63). *Mother’s education level* (NOR) was measured by asking the mothers to indicate their highest level of education on a five point scale ranging from 1, “9 year primary school or less” to 5, “More than 4 years at college or university” and by mother report (AUS) on a three point scale of “Less than secondary school (Year 12)” (1), “Completed secondary school (Year 12)” (2) to “Completed post-secondary education” (3). *Poor family management* (NOR) was assessed using adolescent-report on the short version of the “Keeping tabs” questionnaire developed for the NICHD Study of Early Child Care and Youth Development (SECCYD). The 6-items are questions regarding their parents’ supervision and monitoring (i.e. “how much does a parent know about…”; “who you spend time with?”, “…how you spend your money?”, “…where you go after school?”). Responses ranged from 1 to 4 (“knows everything” to “doesn’t know at all”) and indicate the extent to which the parent is thought to know about different aspects of the child’s whereabouts and day to day activates (NOR, Cronbach’s alpha = .85). In AUS, poor family management was measured by a nine item scale asking adolescents’ agreement to statements such as “My parents ask if I’ve gotten my homework done”, “The rules in my family are clear”, “If you skipped school without your parents’ permission, would you be caught by your parents?”. Response options were: NO! (4), no (3), yes (2), YES! (1) (AUS, Cronbach’s alpha = 0.77). *Alcohol and drug use in the family* was defined to have occurred if the adolescents reported having experienced that “one of the people closest to me uses too much alcohol, pills or other drugs” one or more times during the past 12 months at t5 and/or t6 (0 = No, 1 = Yes) (NOR) and "Has anyone in your family ever had a severe alcohol or drug problem?" (0 = No, 1 = Yes) (AUS). Past year *conduct problems* was assessed by a sum score of 19 adolescent-report items taken from three Scandinavian scales of antisocial behaviour (NOR). The items covered stealing, verbally and physically aggressive behaviours, loitering, vandalism, and questions about carrying weapons. The construction of the scale is described in detail elsewhere [53]. The answers were given on an ordinal frequency scale and for this study transformed to a dichotomous (yes/no) variable, creating a variable ranging from 0 to 18 (Cronbach’s alpha = .84). In the AUS sample, *conduct problems* were measured with nine items covering stealing, physically aggressive behaviour, suspension from school, arrests and questions about carrying weapons, selling drugs and being drunk or high at school. The response options ranged from “never” (1) to “40+ times” (8), (Cronbach’s alpha = 0.64). *Early sexual intercourse* was assessed by one item asking if the adolescents had ever had sexual intercourse (yes/no) (NOR and AUS). *Smoking* was assessed by a single item asking if adolescents had smoked at least once in their life (yes/no) (NOR and AUS). *Friends’ substance use* was assessed by adolescent report on how many of their most important friends; “drinks alcohol approximately once a week”, “have tried hashish, marijuana or other illegal drugs” (NOR) and (in the past 12 months) “how many of your best friends have”; “tried alcohol (like beer, wine, or liquor/spirits) when their parents didn’t know?”, “used marijuana (pot, weed, grass)?”, “used other illegal drugs (like cocaine, heroin, LSD/acid, or amphetamine/speed)?” (AUS). A dummy variable was constructed contrasting those who reported at least one friend with substance use experiences with all others. *Gender* and the *age* of the child at the time of responding to the survey were assessed. Gender was dummy-coded (female coded 0 and male coded 1) (NOR and AUS).

### Statistical analysis

The relationships of adolescent EOD and EOE with hazardous drinking in late adolescence/young adulthood were examined by means of a modified Poisson regression approach where Poisson regression with robust error variances were estimated. This approach has been recommended instead of binary logistic regression analysis because prevalence ratios (PR) or risk ratios are obtained, thereby providing a more intuitive estimate of the association between predictors and outcomes than odds ratios from logistic regression models [54, 55]. Moreover, Poisson regressions can easily be conducted without difficulties converging. In the present study, the two dummy variables for EOD and EOE were simultaneously included as predictors of hazardous drinking (AUDIT ≥8) in modified Poisson regression analyses together with age and gender. Moreover, a series of Poisson regression analyses was performed to identify associations between each potential confounder with hazardous drinking, controlling for age and gender. Finally, EOD, EOE and all potential confounders were included simultaneously in Poisson regression analyses to examine the unique associations of adolescent EOD and EOE with late adolescent/young adult hazardous drinking. To examine the robustness of our results, we re-ran all analyses by using logistic models. All continuous predictor variables were standardized. Results thus indicate the change in the outcome associated with one standard deviation change in the predictor.

The overall proportions of missing data among those who participated was low. In the NOR sample, the variable with highest level of missing was hyperactivity (4.3%), with proportions of missingness varying from 0.3 to 1.8% for the remaining variables. In the AUS sample, early sexual intercourse (26.4%), and mother’s education (7.1%) showed rather high proportions of missingness, whereas the remaining variables had between 0.3 and 2.5% missing data. Multiple imputation (MI) was used to handle missing data in the predictor and confounder variables, thereby providing missing data routines that are considered the state of art and to be appropriate under missing at random (MAR) conditions [56]. As recommended, 20 complete datasets were created by imputation, incorporating all variables of interest [56]. All analysis and MI were conducted separately for the Norwegian and Australian sample. To test the robustness of our results, all analyses were additionally re-run by handling missing data by listwise deletion.

## Results

Table 1 provides the descriptive statistics for the study variables. As shown in the table, a total of 60.2% (NOR) and 54.8% (AUS) of the adolescents in the study were girls. At the time of the study, 13.4% of the Australian adolescents and 10.3% of the Norwegian adolescents’ reported alcohol and drug use in the family.

**Table 1:** Descriptives of study variables

	Mean	Standard deviation
*Norway (n = 329)*		
Girls (a), %	60.2	
Age (m)	14.58	0.31
Hyperactivity (a)	1.13	0.53
Conduct problems (a)	2.60	2.87
Early sexual intercourse (a), %	8.3	
Smoking (a), %	25.4	
Friends’ substance use (a), %	43.3	
Mother’s education (m)		
Primary school (9 years or less), %	3.4	
Secondary school (1–3 years), %	37.5	
Higher education (≤4 years), %	25.7	
Higher education (> 4 years), %	33.4	
Poor family management (a)	1.85	0.58
Alcohol and drug use in the family (a), %	10.3	
*Australia (n = 786)*		
Girls (a), %	54.8	
Age (m)	14.89	0.38
Impulsivity (a)	2.06	0.53
Conduct problems (a)	1.12	0.30
Early sexual intercourse (a), %	11.1	
Smoking (a), %	53.0	
Friends’ substance use (a), %	80.0	
Mother’s education (m)		
Primary school, %	42.2	
Completed secondary school, %	31.9	
Higher education, %	26.1	
Poor family management (a)	1.86	0.48
Alcohol and drug use in the family (a), %	13.4	

*Note.* Variables are measured differently in the Norwegian and Australian samples and values are therefore not directly comparable

(a) = Adolescent self-report; (m) = Mother’s report

The frequency of drinking behaviours in adolescence and late adolescence/young adulthood for both samples is presented in Table 2. In the AUS sample, over half of the adolescents were classified as EOD, whereas 32.2% were classified as EOE. In the NOR sample, about one quarter of the sample was classified as EOD and 13.4% as EOE. Norwegian late adolescents/young adults reported higher rates of hazardous drinking (46.8%) than the Australian late adolescents/young adults (38.9%), with a slight overlap of the confidence intervals of the proportions. As there was a modification in the wording of one of the ten items in the AUDIT (Item 3) in the NOR sample, we also compared AUDIT scores between the NOR and AUS sample when excluding this item. The difference between the samples did not change substantially (mean score for the AUDIT with 10 items: NOR: 7.59 versus AUS: 7.13; mean score when excluding Item 3: NOR: 6.07 versus AUS: 5.68). A further inspection of single items of the AUDIT revealed that late adolescents/young adults in Australia reported a *higher frequency* of drinking (AUDIT Item 1; 2–3 times a week or more) compared to the Norwegian late adolescents/young adults (AUS: 29.7% versus NOR: 6.5%), while a higher proportion of the Norwegian late adolescents/young adults reported *drinking larger amounts* of alcohol per drinking occasion (AUDIT Item 2; five drinks or more; NOR: 64.2% versus AUS: 36.6%).

**Table 2:** Proportion of early onset drinking (EOD), early onset excessive drinking (EOE), abstinent (14–16 years) and hazardous and non-hazardous drinking in late adolescence/young adulthood (18–25) in the Norwegian and Australian sample

	Norway			Australia		
	*n*	%	95% CI	*n*	%	95% CI
*Alcohol use in adolescence*						
EOD	84	25.5	20.8–30.2	442	56.2	52.7–59.7
EOE	44	13.4	9.7–17.1	253	32.2	28.9–35.5
Abstinent	201	61.1	55.8–66.4	91	11.6	9.4–13.8
*Alcohol use in late adolescence/young adulthood*						
Non-hazardous drinking (AUDIT < 8)	173	53.2	47.8–58.6	477	61.1	57.7–64.5
Hazardous drinking (AUDIT ≥8)	152	46.8	41.4–52.2	304	38.9	35.5–42.3

Note. EOD, EOE and abstinence are measured differently in the Norwegian and Australian samples and prevalences are therefore not directly comparable

95% CI = 95% confidence interval of percentage estimate

*AUDIT* Alcohol Use Disorders Identification Test

Modified Poisson regression analyses showed a statistically significant association of both EOD and EOE with hazardous drinking while adjusting for gender and age (Table 3, Model 1). EOE was a stronger predictor than EOD of hazardous drinking in both the NOR and the AUS sample: Prevalence ratios indicated a two to three times higher risk of hazardous drinking among those with EOE, compared to those with no early drinking experiences. EOD was also significantly associated with an increasing risk of hazardous drinking, with an about two-fold increased risk of reporting hazardous drinking, compared to abstinence. Among the possible confounding variables, significant relationships between predictors and outcome were revealed in the individual and the family domain for both samples.

**Table 3:** Results of modified Poisson regressions predicting hazardous drinking (AUDIT ≥8)

	Hazardous drinking		Hazardous drinking	
	(AUDIT ≥8) Norway		(AUDIT ≥8) Australia	
	Model 1^a^	Model 2^b^	Model 1^a^	Model 2^b^
	PR (95% CI)	PR (95% CI)	PR (95% CI)	PR (95% CI)
*Individual variables*				
EOD relative to abstinent (a)	1.78 (1.39–2.30)***	1.63 (1.25–2.13)***	2.18 (1.35–3.51)**	1.69 (1.03–2.78)*
EOE relative to abstinent (a)	2.10 (1.52–2.66)***	1.76 (1.19–2.60)**	2.97 (1.84–4.78)***	1.89 (1.12–3.16)*
Impulsivity (a) (z)	–	–	1.18 (1.09–1.29)***	1.07 (0.97–1.17)
Hyperactivity (a) (z)	1.26 (1.13–1.42)***	1.17 (1.03–1.33)*	–	–
Conduct problems (a) (z)	1.24 (1.14–1.34)***	1.11 (0.97–1.26)	1.10 (1.04–1.17)**	1.00 (0.93–1.07)
Early sexual intercourse (a)	1.16 (0.79–1.71)	0.72 (0.48–1.08)	1.31 (1.01–1.70)*	1.06 (0.79–1.43)
Smoking (a)	1.28 (1.01–1.63)*	0.92 (0.71–2.18)	1.66 (1.36–1.99)***	1.31 (1.06–1.61)*
Friends’ substance use (a)	1.31 (1.04–1.65)*	0.90 (0.69–1.19)	1.72 (1.28–2.33)***	1.24 (0.89–1.72)
Gender (girl = 0, boy =1) (a)	1.37 (1.09–1.72)**	1.36 (1.07–1.71)*	1.63 (1.36–1.95)***	1.59 (1.33–1.89)***
Age (m) (z)	1.00 (0.89–1.13)	0.98 (0.88–1.00)	1.00 (0.93–1.10)	0.92 (0.92–1.08)
*Family variables*				
Mother’s education (m) (z)	1.03 (0.91–1.16)	1.03 (0.92–1.16)	1.03 (0.94–1.13)	1.06 (0.97–1.16)
Poor family management (a) (z)	1.20 (1.07–1.34)**	1.02 (0.90–1.17)	1.24 (1.15–1.35)***	1.11 (1.01–1.22)*
Alcohol and drug use in the family (a)	1.51 (1.14–2.01)**	1.13 (0.82–1.53)	1.09 (0.85–1.39)	0.89 (0.69–1.15)

*Note.* Variables are measured differently in the Norwegian and Australian samples and values are therefore not directly comparable

*P < 0.05; **P < 0.01; ***P < 0.001. PR = Prevalence Ratio; 95% CI = 95% confidence interval of PR

(a) = adolescent self-report; (m) = mother’s report; (z) = standardized variables

^a^Separate analyses are conducted for each predictor, with control for age and gender. PR of age and gender are not controlled for covariates

^b^All predictor variables are included simultaneously

When conducting Poisson regression analyses with adjustment for all confounders, EOE, although somewhat reduced, remained a significant predictor of hazardous drinking in both samples and prevalence ratios remained marginally higher than for the relationship between EOD and the outcome (see Table 3, Model 2). Likewise, the association between EOD and hazardous drinking remained statistically significant in both samples, even though prevalence ratios were somewhat reduced when including covariates. To further examine the role of EOD and EOE relative to each other, we conducted a new set of analysis where the dummy variables for early onset of drinking behaviour were recoded to set EOD as the reference group. We could thus test whether EOD increased the risk of hazardous drinking compared to EOE, when adjusting for all covariates, but no significant difference between EOD and EOE was found (NOR: PR = 1.08, 95% CI = 0.78–1.50; AUS: PR = 1.11, 95% CI = 0.92–1.35). To explore potential differences in the predictive potential of EOD versus EOE in greater detail, a new set of regression analyses was conducted, where any form for early use (including both drinking moderate amounts of alcohol and drinking excessively) was included as one dummy variable, together with all covariates. Comparable to results where EOD and EOE were included as separate variables in the regression analyses, any form for early alcohol use was significantly related to hazardous drinking in both countries (NOR: PR = 1.65, 95% CI = 1.27–2.14; AUS: PR = 1.72, 95% CI = 1.04–2.82). Moreover, to examine whether EOE predicted later hazardous drinking over and above any form of early use, EOE was included as an additional predictor in the regression equations. In line with the previous findings, results showed early use to significantly predict hazardous drinking (NOR: PR = 1.63, 95% CI =1.25–2.13; AUS: PR = 1.69, 95% CI = 1.03–2.78), whereas EOE did not significantly increase the risk of hazardous drinking over and above any early use (NOR: PR = 1.08, 95% CI = 0.77–1.49; AUS: PR = 1.11, 95% CI = 0.92–1.35).

Few significant relationships were identified among the possible confounding variables in the multivariate analysis (see Table 3, Model 2). In the NOR sample, only hyperactivity and male gender significantly increased the risk of hazardous drinking. In the AUS sample poor family management, smoking and male gender remained as statistically significant predictors of hazardous drinking. We also examined whether the relationship between early drinking behaviours and hazardous drinking was gender-specific, but we found no significant interactions.

Finally, all regression analyses were re-run by handling missing data by listwise deletion and results were compared to analyses when MI was used. The analyses showed no substantial differences in the results for the two ways of handling missing data. Moreover, as another robustness check, all modified Poisson models were re-ran by using logistic regressions. The results showed similar results, but with somewhat higher odds ratios compared to prevalence ratios. Such a difference was expected, as odds ratios in general yield higher values than prevalence rates, particularly when highly prevalent phenomena, such as alcohol use, are investigated [55].

## Discussion

This study used prospective, cross-national data to examine the relationship of EOD and EOE with hazardous drinking. The results showed both EOD and EOE in adolescence to be related to an increased risk for alcohol-related problems in late adolescence/young adulthood, with a somewhat stronger association for EOE than EOD. Moreover, the associations were reduced, but remained statistically significant when controlling for a comprehensive number of potential confounders, including risk factors such as conduct problems, alcohol and drug use in the family and friends’ substance use. Even though most risk factors were operationalized somewhat differently in the two samples, the same pattern of findings was identified in both NOR and AUS – two countries with different alcohol policies and drinking cultures.

Our findings support previous longitudinal studies demonstrating that EOD/EOE is related to a high risk alcohol consumption pattern [7, 11, 14, 28–30, 33, 57]. Moreover, the marked attenuation of the relationship between EOD/EOE and alcohol-related problems when including potential confounders is also in accordance with previous empirical studies (for a review, see [18]). However, somewhat surprisingly, the association between early drinking behaviours and hazardous drinking remained statistically significant, even after control for a comprehensive number of possible confounders. Our results thus indicate that adolescents with EOE/EOD are two to three times more likely to be high risk drinkers in late adolescence/young adulthood. Such findings are in contrast to previous prospective studies in which associations between early drinking behaviour and later high risk drinking patterns were completely explained by relevant covariates [16, 31, 32], but well in line with the findings of Buchmann et al. [4] and Irons et al. [11].

The divergent findings may be explained by variations in the age at which problematic drinking patterns were assessed. Typically, in studies assessing adult outcomes at ages 25 and above [16, 31, 32], associations between early drinking behaviour and later high risk drinking were not detected. In contrast, the study of Buchmann et al. [4], Irons et al. [11], and the present study, which detected associations between early drinking behaviour and later high risk drinking assessed later age drinking at 18 to 25 years. This pattern of findings may suggest that the effect of EOD/EOE on later high risk drinking may be particularly potent in late adolescence/young adulthood.

Excessive drinking typically reaches a peak in late adolescence/young adulthood, and then gradually decreases with increasing age, accompanied by changing roles and obligations as one moves through different life phases [58–60]. A high risk drinking pattern, in the late adolescence/young adult life phase, where excessive drinking is “normative” and highly prevalent, may to a larger degree be based on social and environmental factors – including early alcohol exposure, relative to later high risk drinking. Maintenance of an excessive drinking pattern throughout adulthood is typically restricted to a smaller group of individuals characterized by more severe and long-lasting problems [60]. This drinking behaviour may be better understood in the framework of underlying genetic liability, than in light of social and environmental factors. Such a notion is supported by several genetically informed studies showing that heritability estimates increase with increasing age of the individuals and increasing severity of alcohol-related problems [61, 62]. EOD may become a non-significant predictor of adult alcohol problems when adjusting for factors thought to reflect genetic transmission or vulnerability, such as externalizing behaviours and parental substance use. In contrast, early drinking experiences in themselves may function as an early transition to a drinking culture, and may thus increase the risk for hazardous drinking in social contexts, particularly in late adolescence/young adulthood where such behaviour is considered normative.

The scientific community has over the past couple of decades discussed whether more than just a few sips of alcohol per se or having consumed more substantial amounts of alcohol at an early age are related to adverse outcomes (e.g., [12, 33, 63]). The present study shows rather minor differences in how EOD versus EOE prospectively predicts later hazardous drinking behaviour. There could be several explanations for such a finding. First, early drinking behaviours, regardless of level of alcohol consumption, may be indicators of the same proneness to risk-taking behaviours, including drinking. However, previous analyses of predictors of EOD and EOE in the NOR sample suggest that adolescents with EOD may be less inclined to take risks than adolescents with EOE, as lower levels of shyness, conduct problems and high levels of risk exposure in friendship networks were unrelated to EOD, but prospectively predicted EOE [64]. Thus, adolescents with EOE are in fact more likely to show characteristics indicating higher proneness to risk taking behaviour than EOD adolescents.

Alternatively, drawing on neurobiological and psychosocial development literature, there may be similar biological mechanisms whereby alcohol exposure leads to later alcohol-related problems [35, 36]. Based on our findings, we cannot dismiss such a biological basis, although further investigations of these relationships with a more fine-grained measure of different levels of early alcohol exposure is warranted. Furthermore, this hypothesis would be strengthened if the relationship between early onset and alcohol-related problems more consistently demonstrated a robust relationship across different studies. Our findings are more in line with an understanding of early onset, regardless of level consumed, as an introduction to a “wet” environment, enabling a progression to a hazardous drinking pattern. Interestingly, similar patterns in the relationship between early onset and risk of alcohol-related problems were identified in both the NOR and AUS samples, despite the very different proportions of adolescent drinking. The results indicate that early alcohol use seems be cross-nationally invariant and to a lesser degree is influenced by differences in cultural or social norms and national alcohol policies. Furthermore, rates of hazardous drinking were higher in the NOR sample despite the much greater adolescent abstinence. This finding indicates that EOD/EOE cannot fully explain hazardous drinking in late adolescence/young adulthood and that there are likely to be important contextual correlates that affect this behaviour.

### Strengths and limitations

By using prospective studies, we minimized the potential bias caused by retrospective recall and forward telescoping that are often noted as major limitations in the literature on early onset of alcohol use [65]. The inclusion of a wide range of potential confounding factors, consideration of the independent importance of different early drinking behaviours and the use of two samples from different cultural contexts, make this study an important contribution to the literature on consequences of early drinking patterns. However, several considerations are important to note. First, the measures of early drinking behaviours give us no indication of the actual volume of alcohol consumed, although the better differentiation of early drinking experiences into EOD and EOE improves upon previous studies. Second, the NOR and AUS studies were designed as two separate studies. Consequently, the predictor variables and covariates were assessed differently in the Norwegian and the Australian samples and are not directly comparable. For example, the higher prevalence of early adolescent drinking in the AUS sample may not necessarily reflect different drinking patterns between the countries, because drinking was assessed differently in the two samples. Another limitation is that the two samples differed in the age when hazardous drinking was assessed in “late adolescence/young adulthood”. While the Norwegian sample had just reached the legal drinking age (mean age 18.9 years), the Australian participants were on average 22.9 years old. Such differences may explain different rates of hazardous drinking in the two samples, and it is possible that the Australian participants have “settled down” somewhat as compared to the Norwegian adolescents, who may just have moved away from home and take full advantage of their new won freedom. Still, the age at which most adolescents start to drink is very similar in many Western countries. Around 50% or more of 15–16 year olds have initiated alcohol use [66]. As such, many adolescents have several years of experience with alcohol before reaching young adulthood. Despite different measures and age ranges, the patterns identified across samples were similar, indicating a robust association across countries with different alcohol policies and drinking cultures. Third, even though a comprehensive number of potential confounders were included, we did not have access to information about parents’ drinking patterns, physiological sensitivity to the effects of alcohol or genetics. The relation between early drinking behaviour and hazardous drinking could thus be caused by unmeasured common risk factors. Another concern is that information about the adolescent drinking context was not available in this study. Fourth, some measures had somewhat low internal reliability. Particularly, the three item impulsivity scale used in the Australian sample displayed low Cronbach’s alpha, with a value of .57. Pearson’s correlations, which the Cronbach’s alpha is based on, holds an assumption of normally distributed continuous variables. The items in the impulsivity scale are on an ordinal scale, with few response categories, and a slightly skewed distribution. As such, calculation of alpha based on Pearson’s correlation may lead to underestimation of the true association. In such cases, alternative strategies to estimate reliability have been suggested, such as calculating Cronbach’s alpha based on polychoric correlations, providing a more accurate estimate of the relationship between the items on the scale [67]. In our case, such an alternative estimation strategy increased internal consistency somewhat to α = .63. Even though it is common to find low alphas in scales consisting of few items, we cannot rule out that a more reliable assessment of impulsivity could have affected the results in our study. Fifth, AUDIT scores for the adolescent waves were not available, thereby not providing us with the possibility to examine alcohol-related problems in greater detail or to account for already existing problems when examining the relationship between early onset of alcohol use and later alcohol-related problems. Lastly, the substantial attrition rate in the NOR sample is a major limitation of the study, as only data from 30.4% of the originally invited families were available at both time points of interest. Although attrition rates of 50% and more are not uncommon in longitudinal studies [68], such high attrition rates may be a source of bias. Of particular concern is the fact that attrition in the NOR sample was predicted by low maternal education level and male gender, resulting in an overrepresentation of girls and adolescents whose mothers have higher levels of education. This may represent a threat to the representativeness, and our study provides limited knowledge about how early drinking behaviours are related to later alcohol outcomes in adolescents whose mothers have a low level of education. Moreover, the selective drop-out warrants caution when interpreting the results regarding gender, maternal education and family variables, even though attrition and simulation studies based on the NOR sample have shown that selective attrition leads only to biased estimates of means of variables; estimates of associations between variables were not affected even with selective attrition and high attrition rates [69]. Still, research on these associations in low socio-economic strata will be of importance in future studies. The AUS sample is a Victoria sample, and so may be limited in its generalisability to the whole country.

## Conclusion

In summary, this study used prospective, cross-national data to examine the relationship of EOD and EOE with hazardous drinking in late adolescence/young adulthood. Our findings indicate that early drinking behaviour, regardless of level, is an indicator of alcohol-related problems in late adolescence/young adulthood even when controlling for a variety of covariates. This pattern is consistent across samples drawn from two countries with different prevention policies and drinking cultures. Our findings are in contrast to previous research on older adults, where no association between adolescent drinking and later alcohol problems was found when controlling for covariates. The divergence in findings may suggest that the impact of EOD/EOE is limited to the late adolescent and young adult period. We suggest that EOD and EOE play a role first and foremost as an introduction to a drinking culture enabling a progression to a hazardous drinking pattern. Efforts to reduce early age drinking and reduce the progression from experimental/minor alcohol use to instances of EOE may have some impact on the risk of alcohol-related problems in late adolescence/young adulthood. Future research should examine the role of early drinking behaviours and indicators of alcohol-related problems in different life phases.

## Data Availability

The TOPP study provides access to data for all members of the TOPP study. Data for external researchers can only be made available on request, provided that the TOPP-research group has available resources to facilitate and administer collaboration. The IYDS study provides limited access to approved collaborators. Core researchers of the IYDS have permission to use the data, but data for external researchers can only be made available on request provided that the IYDS principal investigators have approved the collaboration.

## Abbreviations

AUDIT: Alcohol Use Identification Test
AUS: Australia/Australian
CI: Confidence interval
EOD: Early onset of drinking
EOE: Early onset of excessive drinking
NOR: Norway/Norwegian
PR: Prevalence ratios
